# Efficient Transient Expression of Plasmid DNA Using Poly (2-(*N*,*N*-Dimethylamino) Ethyl Methacrylate) in Plant Cells

**DOI:** 10.3389/fbioe.2022.805996

**Published:** 2022-02-22

**Authors:** Zishuai An, Bing Cao, Junzhe Zhang, Baihong Zhang, Chengqian Zhou, Xianglong Hu, Wenli Chen

**Affiliations:** ^1^ MOE Key Laboratory of Laser Life Science and Institute of Laser Life Science, College of Biophotonics, South China Normal University, Guangzhou, China; ^2^ Guangdong Provincial Key Laboratory of Laser Life Science, College of Biophotonics, South China Normal University, Guangzhou, China; ^3^ Guangzhou Key Laboratory of Spectral Analysis and Functional Probes, College of Biophotonics, South China Normal University, Guangzhou, China; ^4^ Neuroscience Laboratory, Hugo Moser Research Institute at Kennedy Krieger, Baltimore, MD, United States

**Keywords:** poly (2-(N, N-dimethylamino) ethyl methacrylate) (PDMAEMA), polyethylenimine (PEI), plant cells, gene delivery, gene transfection

## Abstract

Nanomaterials have been widely studied for their potential to become the new generation of nanocarriers in gene transfection, yet it remains still difficult to apply them efficiently and succinctly to plant cells. Poly (2-(*N*,*N*-dimethylamino) ethyl methacrylate) (PDMAEMA), which possesses temperature and pH dual-sensitivity, has largely been applied in animal cells, but it is rarely involved in plant cells. As a proof of concept, PDMAEMA as a gene carrier is incubated with plasmid GFP (pGFP) to explore its transfection ability in plants, and cationic polymer polyethylenimine (PEI) is used as a control. pGFP was efficiently condensed into the nanostructure by electrostatic interactions at an N/P (amino group from cationic polymers/phosphate group from plasmid DNA (pDNA)) ratio of 15; after complexation into nanocarriers, pGFP was protected from endonuclease degradation according to the DNase I digestion assay. After incubation with protoplasts and leaves, GFP was observed with confocal microscopy in plant cells. Western blot experiments confirmed GFP expression at the protein level. Toxicity assay showed PDMAEMA had a lower toxicity than PEI. These results showed that transient expression of pGFP was readily achieved in *Arabidopsis thaliana* and *Nicotiana benthamiana*. Notably, PDMAEMA showed lower cytotoxicity than PEI upon incubation with *Nicotiana benthamiana* leaves. PDMAEMA exhibited great potency for DNA delivery in plant cells. This work provides us with new ideas of more concise and more effective methods for plant transformation.

## Introduction

Great progress has been made in plant biotechnology in the recent years, but it remains still difficult to efficiently perform genetic transformation on plants (Altpeter et al., 2016). Although the agrobacterium-mediated delivery system is the most classic method for plant genetic transformation, it still has defects such as limitation of plant species and low transformation efficiency (Baltes et al., 2017). Biolistic (gene gun) is another plant transformation tool, which can deliver biomolecules into more general plants without species limitation, but it may cause plant tissue damage under high bombardment pressure and require a large amount of DNA to perform an efficient plant transformation (Altpeter et al., 2016). Plant viral vectors such as tobacco mosaic virus can also be used to perform transient expression of the exogenous gene in plants. Viral vectors are compatible with various plant species. However, due to relatively narrow virus–host specificity, different plant species may require different vectors.

In recent years, increasing interests have been focused in biological and biomedical applications of nanomaterials (Mei et al., 2019). Among them, the application of nanomaterials for gene delivery in animal cells has been widely studied (Gregory et al., 2020; Yan et al., 2020). Various delivery platforms such as mesoporous silica nanoparticles (Ding et al., 2020), carbon nanotubes (Cifuentes-Rius et al., 2017), gold nanoparticles (Huo et al., 2014; Ortega-Munoz et al., 2016), quantum dots (Liu et al., 2019), magnetic nanoparticles (Lo Y.-L. et al., 2015; Huang et al., 2019), DNA origami (Liu et al., 2018), nanodroplets (Zhang et al., 2019; Cao et al., 2020), and polymers (Chu et al., 2020; Guo et al., 2020; Wang et al., 2020; Wang Z. et al., 2021) can load drugs or other active biomolecules and deliver them to target sites due to their relatively small size, special chemical composition, or functionalized surface structure. Among these many nanomaterials, cationic polymers have attracted more and more attention in molecular delivery due to their advantages of easy synthesis, high stability, low toxicity, low immunogenicity, and compatibility with larger molecular payloads (Yin et al., 2013; Lo C.-W. et al., 2015; Ding et al., 2021). Positively charged cationic polymers can interact with negatively charged DNA by electrostatic interactions and condense DNA into a compact complex in nanoscale (Lo C.-W. et al., 2015). The charge of the complex remains positive and makes it easier to internalize into cells based on the interaction with the negatively charged cell membranes (Lo C.-W. et al., 2015; Demirer et al., 2019). Poly (2-(*N,N*-dimethylamino) ethyl methacrylate) (PDMAEMA) and polyethylenimine (PEI) are two typical cationic polymers with a high density of positively charged amine groups, and have both been used for DNA delivery in animal cells (Wu et al., 2011; Yu et al., 2012; Lo C.-W. et al., 2015; Guo et al., 2021; Richter et al., 2021). Yet, their applications in plant cells are rarely investigated. Hence, it is meaningful to interrogate their potency for plants, such as the controlled release of agrochemicals and target-specific delivery of biomolecules (Faraz et al., 2019; Bijali and Acharya, 2020; Wang W. et al., 2021). PEI shows a relatively high toxicity than other cationic polymers including PDMAEMA in animal cells (Lo C.-W. et al., 2015). Compared with human cancer therapy, there are few studies on the application of nanotechnology in plants. In agriculture field, applications of nanomaterials have been found to efficiently resist environmental stress and improve the efficiency of agrochemicals, including fertilizers and pesticides in an environment-friendly way (Khot et al., 2012; Shang et al., 2019; Fincheira et al., 2020). Mesoporous silica nanoparticles have shown great potential to deliver an exogenous gene into intact *Arabidopsis thaliana* roots and protoplasts without any mechanical aids (Torney et al., 2007; Chang et al., 2013). Additionally, carbon nanotubes with high aspect ratios can efficiently deliver plasmid DNAs (35S–GFP–NOS and UBQ10–GFP–NOS) to several mature plants (Demirer et al., 2019; Kwak et al., 2019). However, there is still a need to develop new delivery methods with good transfection efficiency, good biocompatibility, low toxicity, and immunogenicity in plant cells (Keles et al., 2016).

In our previous study, functioned gold nanoparticles were used to carry a small interfering RNA and successfully silenced a target gene (*NPR1*) in *Arabidopsis thaliana* (Lei et al., 2020). To further explore the transfection behavior of PDMAEMA in plant cells, we combine plasmid GFP (pGFP) as a reporter gene with PDMAEMA and incubated them with protoplasts and leaves to transfect plant cells. GFP fluorescence was successfully observed with confocal microscopy in plant cells. GFP expressions were also detected in the protein level. We managed to transiently transfect plant cells using PDMAEMA, which laid the foundation for the genetic transformation of plants.

## Materials and Methods

### Materials

Plasmid DNA (pDNA) pBI221-GFP (Supplementary Figure S1) was purified using a HiPure Plasmid EF Maxi Kit (Magen, Guangzhou, China) for all experiments in this study. The concentration and purity of pGFP were determined by the absorbance ratio at OD_260_/OD_280_ using a Nano Drop 2000 (Thermo Scientific). Cellulase R-10 (MX7352, Yakult Japan) and MacerozymeR-10 (DH188-2, Dingguo, China) were used for protoplast extraction. The following chemicals were purchased from Sigma-Aldrich: 4-cyano-4-(phenylcarbonothioylthio) pentanoate (CPADB), 2-(dimethylamino) ethyl methacrylate (DMAEMA, 99%) sodium chloride, calcium chloride dehydrate, 2-(N-morpholino) ethanesulfonic acid (MES), D-mannitol, potassium chloride, magnesium chloride hexahydrate, polyethylene glycol (4,000), and polyethylenimine (branched, 25 kDa). 2, 2′-Azobis (2-methylpropionitrile) (AIBN) was obtained from Acros chemicals. 1,4-Dioxane was purchased from Sinopharm Chemical Reagent Co. Ltd. Water used in the study was deionized with a Milli-QSP reagent water system (Millipore).

The seeds of *Arabidopsis thaliana* and *Nicotiana benthamiana* were germinated in pots with a mixture of soil and vermiculite at a ratio of 4:1. The two plants were both grown in the growth chamber (16 h light at 23°C/8 h dark at 23°C). The light intensity was approximately 120 μmol photons m^−2^s^−1^, and the relative humidity was about 82%.

## Methods

### Synthesis of PDMAEMA

The synthesis of PDMAEMA was referred to the previous report (Hu et al., 2014; Cao et al., 2020; Xiao et al., 2020). The chain transfer agent, 4-cyano-4-(phenylcarbonothioylthio) pentanoate (CPADB, 73.3 mg, 0.262 mmol), 2-(dimethylamino) ethyl methacrylate (DMAEMA, 1,450 mg, 9.22 mmol), and 2, 2′-azobis (2-methylpropionitrile) (AIBN, 8.6 mg, 0.052 mmol) were mixed and charged into a glass ampoule containing 1,4-dioxane (1.375 mL). The ampoule was degassed via three freeze–pump–thaw cycles and flame-sealed under vacuum. Then, the glass ampoule was immersed into an oil bath (70°C) to start polymerization. After 12 h, the ampoule was quenched into liquid nitrogen to terminate the polymerization. The mixture was precipitated into an excess of petroleum ether to generate red residues; the residues were dissolved in dichloromethane and precipitated into petroleum ether. After three cycles of dissolution–precipitation, the final product was dried in a vacuum oven overnight at room temperature, yielding a red solid (1,085 mg, yield: 71.2%). The degree of polymerization of DMAEMA was determined to be ∼37 based on the ^1^H NMR analysis (Supplementary Figure S2).

#### Preparation of PDMAEMA + DNA and PEI + DNA Complexes

Gel retardation assay was performed to determine the N/P ratio of the cationic polymer and DNA (Luo et al., 2011). The complexes of pGFP and polymer were freshly prepared before experiments. The N/P ratio of the complex was counted according to the molar ratio of the amino group from the cationic polymer relative to the phosphate group from pDNA. The solution of plasmid was added to the solution of polymer by gradually increasing PDMAEMA or PEI and the same amount of pGFP. N/P ratios were 1:1, 2:1, 5:1, 10:1, and 15:1. The mixtures were then incubated at room temperature for 30 min to form stable complexes and were run electrophoresis on a 1% agarose gel.

#### Measurements of Particle Size

A total of 5 micrograms of DNA (1,600 ng/μL) were mixed with PDMAEMA (1 mg/mL) or PEI (1 mg/mL) at the fixed N/P ratio of 15. The formed complexes were diluted in 10 mM MgCl_2_/MES (pH 5.7) to a final volume of 800 μL. The particle sizes of PDMAEMA + DNA and PEI + DNA complexes were evaluated using the Nano-ZS (Malvern, U.K.). The data are calculated as the means of three measurements.

#### DNase I Protection Assay

DNase I protection assay was performed as described in reference by Luo et al., (2011) with some modifications to investigate the ability of polymers to protect DNA against endonuclease degradation. The samples of PDMAEMA + DNA (N/P 4.38:1) and PEI + DNA (N/P 2.6:1) complexes were freshly prepared. After incubating the complexes for 30 min at room temperature, samples were treated with DNase I at 37 °C for 10 min, followed by denaturation of DNase I at 65 °C for 10 min, and 2 μL of sodium dodecyl sulfate (SDS, 1% w/v, final concentration 0.1%) was added, and the samples were incubated for 2 h at 37 °C to completely dissociate DNA from the complexes. The samples of naked DNA with or without DNase I treatment were used as positive or negative controls, respectively. The DNA dissociated from PDMAEMA or PEI after DNase I treatment was run on a 1% agarose gel.

#### Protoplast Isolation From *Arabidopsis thaliana* Leaves

Protoplasts were isolated from the leaves of wild-type *Arabidopsis thaliana* as described by Yoo et al., (2007). In brief, epidermis-removed leaves by adhesive tape were immersed in 15 mL of enzyme solution (1.5% cellulase R-10, 0.75% Macerozyme R-10, 0.5 M mannitol, 10 mM MES with pH 5.7, 10 mM CaCl_2_, and 0.1% BSA), then incubated at 25 °C for 3 h in the dark with stirring gently. The undigested leaf tissue was removed by filtration with a 75-μm nylon mesh, and then 10 mL W5 solution (1.54 mM NaCl, 125 mM CaCl_2_, 5 mM KCl, 2 mM MES with pH 5.7, and 5 mM glucose) was added, followed by centrifugation at 60 rcf for 5 min at 4 °C. The pelleted protoplasts were resuspended in W5 solution with a pH of 5.7, which has similar osmolality and pH to those of the protoplasts. The isolated protoplasts are viable on ice for over 24 h. Even then, the freshly isolated protoplasts should be used for gene expression.

#### Protoplast Transformation With PDMAEMA + DNA, PEI + DNA, and PEG/Ca^2+^

Protoplast transfection was performed as described in reference by Demirer et al., (2019) with some modifications. A volume of 100 μL (about 1×10^4^) of isolated protoplasts in W5 solution was added to about 10 μL of PDMAEMA + DNA, PEI + DNA containing 10 μg DNA, or for the control sample containing the same amount of pDNA and mixed well by gently tapping the tube. The mixture was incubated at room temperature for 24 h to ensure sufficient internalization and expression. For PEG/Ca^2+^ transformation, *Arabidopsis* mesophyll protoplasts were added to 110 μL PEG-Ca^2+^ solution (100 mM CaCl_2_, 0.2 M mannitol, and 40% PEG 4000) for 15 min incubation at room temperature. Then, protoplasts were diluted in 220 μL W5 solution, followed by 440 and 880 μL W5 solution to wash off the PEG, and after which the protoplasts were harvested by centrifugation at 60 *g* for 5 min at 4 °C. The pellet was washed twice with W5 solution, resuspended in 100 μL W5 solution, and incubated for 24 h at room temperature in the dark. Confocal laser scanning microscope (CLSM) imaging was performed to detect GFP expression by imaging the protoplasts.

#### Infiltration of Leaves With PDMAEMA + DNA, PEI + DNA, or PDMAEMA + FAM-siRNA_*NPR1*_

Healthy leaves from *Arabidopsis thaliana* (three to four weeks old) and *Nicotiana benthamiana* (3 weeks old) were chosen for experiments. After preparation of PDMAEMA + DNA and PEI + DNA complexes containing 10 μg DNA at a N/P ratio of 15 (total volume 10 μL), it was diluted with 10 mM MgCl_2_/MES with pH 5.7 to a final volume of 200 μL and then infiltrated on the abaxial surface of the leaves using a 1-mL needleless syringe by applying gentle pressure. FAM-siRNA_*NPR1*_ (20 μM) (Lei et al., 2020) was mixed with PDMAEMA (1 mg/mL) at the fixed N/P ratio of 15 and incubated at room temperature for 30 min. The formed complexes were diluted 10 times in 10 mM MgCl_2_/MES (pH 5.7) before infiltration.

#### Confocal Microscope Imaging

The images of *Arabidopsis thaliana* protoplasts and leaves were captured using a confocal laser scanning microscope (Carl Zeiss LSM 880 META, Germany). The captured images were processed with commercial Zen software (Carl Zeiss, Germany). GFP fluorescence was captured with 488 nm laser excitation and 539 nm emission wavelengths. Chlorophyll autofluorescence was captured with 633 nm laser excitation and 691 nm emission wavelengths.

#### Protein Extraction and Western Blot

The protoplasts after incubation with PDMAEMA + DNA or PEI + DNA and the cell culture in the dark were added an equal volume of 2× sample buffer directly at 100 °C for 10 min. For protein extraction of plant leaves, 0.4 g of leaf tissue was ground in liquid nitrogen before adding extraction buffer. The extracts were centrifuged, and the supernatant protein was collected to be denatured in the SDS sample buffer at 100 °C for 10 min. The same amount of total protein for each sample was run on 10% SDS-PAGE gels.

After electrophoresis, protein was transferred onto a polyvinylidene difluoride (PVDF) membrane and then blocked for 2 h with 5% (w/v) skim milk powder in TBS plus 0.1% (v/v) Tween 20 (TBST). For GFP detection, the antibody (JL-8, Monoclonal Antibody, A-6455, WB: 1:1,000, Fisher, Invitrogen, Waltham, MA, USA) directly against GFP protein was diluted in TBST at a ratio of 1:1,000 and incubated with the membrane overnight at 4 °C. The membrane was then washed three times with TBST for 10 min. Goat anti-mouse IgG-HRP (Absin, abs 20001) was then incubated with the membrane for 2 hours at room temperature. The membrane was washed twice for 10 minutes and another one time for 30 minutes with TBST. Clarity Western Substrate (BIO-RAD) was used for chemiluminescent detection of the GFP protein. The detection was performed using a LI-COR Odyssey Infrared Imaging System (Tanon, 5,200, China).

#### Toxicity Assay

To evaluate plant toxicity, the expression of a known gene called *respiratory burst oxidase homolog B* (*NbRboh B*) in *Nicotiana benthamiana* leaves infiltrated with different samples was detected by qPCR analysis (Demirer et al., 2019), and three-week-old leaves of *Nicotiana benthamiana* were infiltrated with buffer (10 mM MgCl_2_/MES with pH 5.7, as a negative control), 1% SDS (as a positive control), PDMAEMA + DNA (N/P ratio of 15:1), and PEI + DNA (N/P ratio of 15:1). The infiltrated leaves were collected respectively at 1 h, 3 h, 6 h, and 12 h post-infiltration. The total RNA was extracted from the collected leaves using Trizol and reverse transcribed into cDNA using HiScript RT SuperMix (Vazyme). qPCR was performed using Hieff qPCR SYBR Green Master Mix (Yeason Biotech). *Elongation factor 1* (*EF1*) was measured as a reference gene in the experiment.

Primers for *NbRboh B* are as follows:

Forward: 5 ′-TTT​CTC​TGA​GGT​TTG​CCA​GCC​ACC​ACC​TAA-3′; Reverse: 5′-GCC​TTC​ATG​TTG​TTG​ACA​ATG​TCT​TTA​ACA-3′.

Primers for *EF1* are as follows:

Forward: 5′-TGG​TGT​CCT​CAA​GCC​TGG​TAT​GGT​TGT-3′; Reverse: 5′- ACGCTTGAGATCCTTAACCGCAACATTCTT-3′(Demirer et al., 2019).

qPCR was run at an annealing temperature of 60 °C with 40 cycles. The fold change of *NbRboh B* expression was normalized with respect to EF1 known as a reference gene. qPCR assay was performed for PDMAEMA + DNA, PEI + DNA, and 1% SDS in triplicate-independent experiments. For each sample, three technical replicates and three biological replicates were performed.

Besides the qPCR analysis, the Fv/Fm ratio is also commonly used to indicate the plant toxicity assay (Demirer et al., 2019). The Fv/Fm ratio represents the variable/maximum fluorescence measurement of the photosystem II in plants. The same 3-week-old leaf of *Nicotiana benthamiana* was infiltrated on four different locations at the abaxial surface with the buffer (10 mM MgCl_2_/MES with pH 5.7) as a negative control and PDMAEMA + DNA (N/P ratio of 15:1), PEI + DNA (N/P ratio of 15:1), or 1% SDS as a positive control. The infiltrated leaves were then incubated for 24 h. Subsequently, the infiltrated leaf was dark-treated for 30 min, and chlorophyll fluorescence-related parameters were measured using the Imaging-PAM Maxi fluorimeter (Walz) to calculate the Fv/Fm ratio.

#### Data Processing and Statistical Analysis

All experiments were repeated at least three times or three parallel replicate samples, and the results were processed by GraphPad Prism 8, and then the *t*-test (*p < 0.05, **p < 0.01, ***p < 0.001, and ****p < 0.0001) was used for statistical analysis. Data were expressed as mean ± SD.

## Results and Discussion

### Characterization of PDMAEMA + DNA and PEI + DNA

**FIGURE 1:**
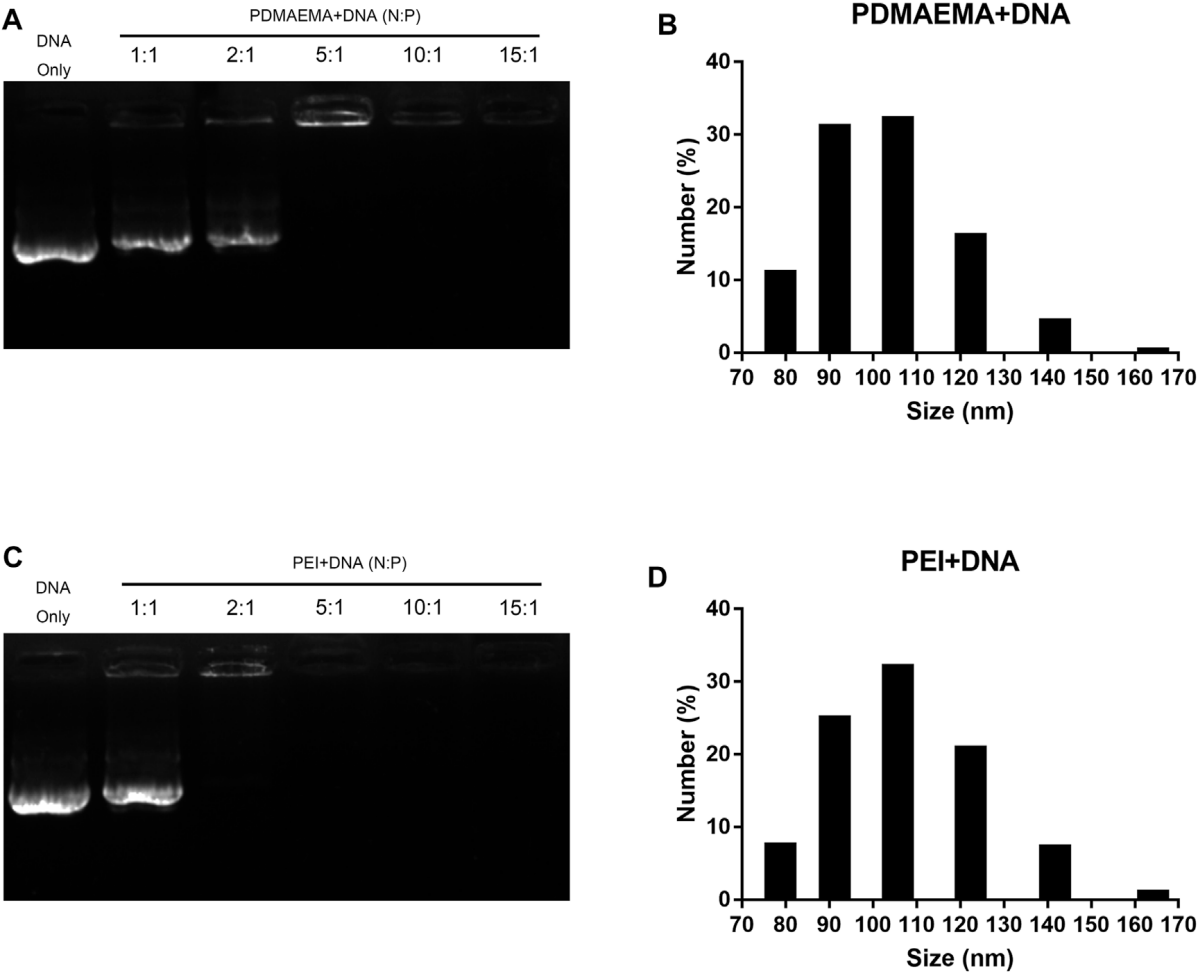
Characterization of PDMAEMA + DNA and PEI + DNA. **(A)** Gel retardation assay of PDMAEMA + DNA (1 μg) at N/P ratios of 1:1, 2:1, 5:1, 10:1, and 15:1. **(B)** Hydrodynamic diameter distributions of PDMAEMA + DNA (5 μg) (N/P ratio 15) determined by dynamic light scattering. **(C)** Gel retardation assay of PEI + DNA (1 μg) at N/P ratios of 1:1, 2:1, 5:1, 10:1, and 15:1. **(D)** Hydrodynamic diameter distributions of PEI + DNA (5 μg) (N/P ratio 15) determined by dynamic light scattering.

Gel electrophoresis shift assay was performed to characterize the interaction and stabilities of the DNA complexes with PDMAEMA or PEI. As shown in Figures 1A,C, two polymers combined with the same amount of DNA (1 μg) at different N/P ratios showed different migration of DNA across the gel relative to DNA only. All bands of the DNA complexes with PDMAEMA or PEI were present at different positions compared to those of free DNA. The amount of the migrated free DNA was reduced with an increasing N/P ratio. In other words, with a low N/P ratio, DNA has not been completely coated with PDMAEMA or PEI, and DNA bands can be seen. When the N/P ratio increased, all DNAs were gradually wrapped to form a stable complex, and the DNA bands were no longer visible. The optimal N/P ratio of PDMAEMA + DNA was 4.38:1 and that of PEI + DNA was 2.6:1 (Supplementary Figures S3A, B). As shown in Figures 1A,C, about 100% of the DNA was captured by PDMAEMA at an N/P ratio of 10 and PEI at an N/P ratio of 5, and thus no free-form plasmid DNA remained in the lane of the gel. Figures 1B,D show the average sizes for complexes of PDMAEMA + DNA and PEI + DNA formed at a constant DNA concentration of 5 μg and at an N/P ratio of 15. The average hydrodynamic diameters of the complexes PDMAEMA + DNA (N/P ratio 15) and PEI + DNA (N/P ratio 15) dispersed in 10 mM MgCl_2_/MES (pH 5.7) were both between 80 and 165 nm, respectively. The most distribution of the diameter of PDMAEMA + DNA and PEI + DNA is 105 nm (Figures 1B,D). However, the size of complexes at a low N/P ratio (4.38:1 for PDMAEMA + DNA and 2.6:1 for PEI + DNA) is larger than that of complexes at a high ratio of 15 (See Figures 1B,D and Supplementary Figures S1B, D). Besides, higher N/P ratios are more conducive to transfection efficiencies (Clamme et al., 2003; Maury et al., 2014; Neuberg and Kichler, 2014). Therefore, an N/P ratio of 15 was chosen for all the transfection experiments in this study.

### PDMAEMA and PEI Protect DNA From DNase I Degradation

**FIGURE 2:**
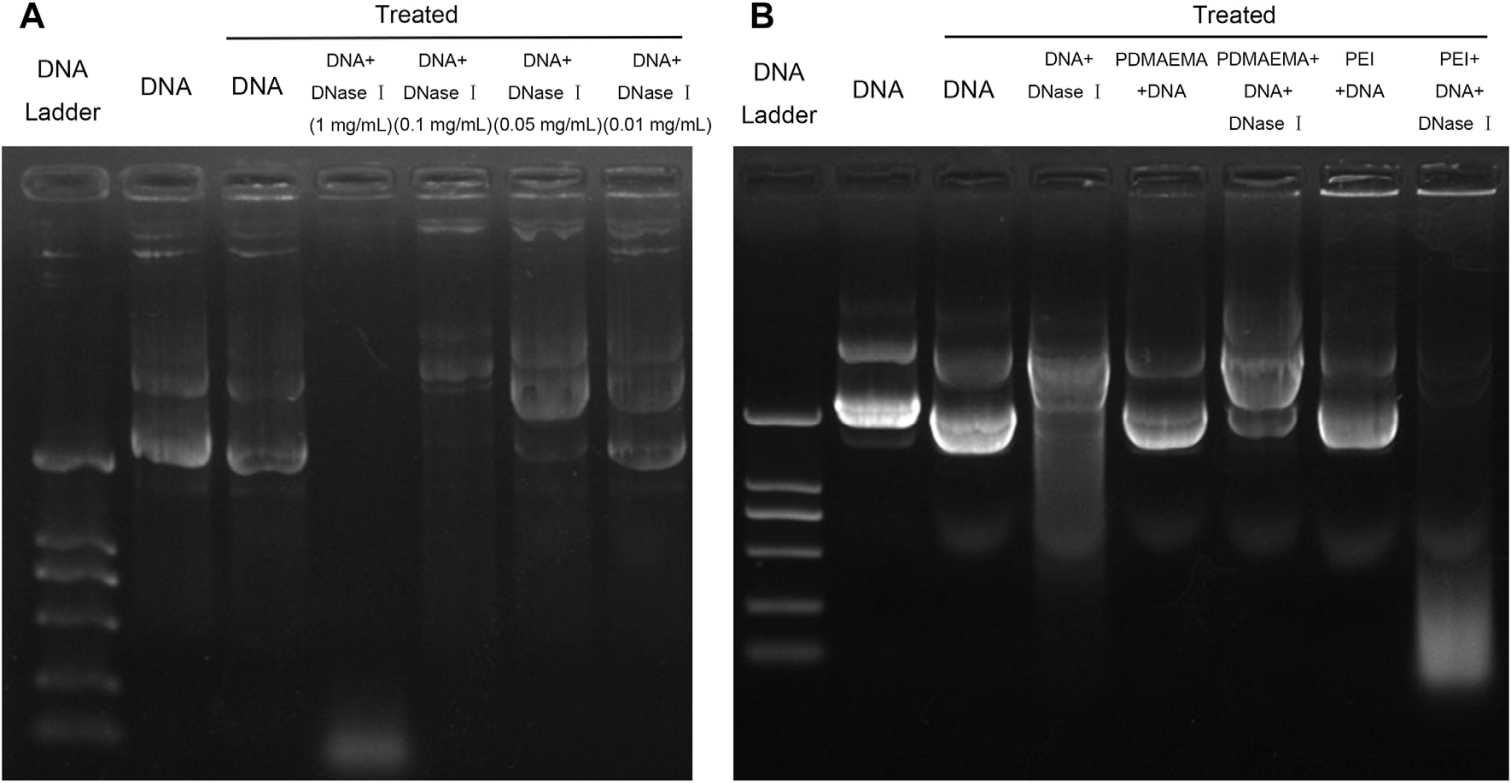
Plasmid DNA protection assay. **(A)** Same amount (1 μg) of pDNA treated by DNase I was run on a 1% agarose gel to determine the optimal DNase I concentration for DNA degradation. **(B)** Agarose gel electrophoresis of DNA, PDMAEMA + DNA (N/P ratio of 4.38:1), and PEI + DNA (N/P ratio of 2.6) incubated with DNase I to evaluate DNA protection against nuclease degradation.

Protection of DNA from nuclease degradation facilitates cell internalization and improves the gene delivery efficiency *in vivo* and *in vitro* (Luo et al., 2011). In consideration of the degradation of nuclease in cells, DNase I was employed to mimic the nuclease in the cell and to test the ability of PDMAEMA and PEI in protecting DNA from nuclease degradation (Lo C.-W. et al., 2015). First, we needed to determine an appropriate concentration of DNase I for this treatment. A decreasing concentration gradient of DNase I was respectively added to the same amount of DNA (1 μg). The different amounts of DNA are shown across the gel with treatment at different concentrations of DNase I (Figure 2A). DNA was slightly decomposed after treatment at 37 °C for 10 min (Lane 3, Figure 2A) compared to the untreated DNA (Lane 2, Figure 2A). A small amount of DNA was degraded after DNase I treatment at concentrations of 0.05 mg/mL and 0.01 mg/mL, but DNA was degraded (Lane 4, Figure 2A) after that of 1 mg/mL. Most of the DNA was degraded after DNase I treatment of 1 mg/mL (Lane 5, Figure 2A). DNase I treatment of 1 mg/mL is excess for DNA degradation. A lane of 0.1 mg/mL DNase I treatment is more optimal than the lane of DNA and DNA treated. Therefore, the optimal concentration (0.1 mg/mL) of DNase I for DNA degradation was determined. The N/P ratio is important for a compact combination of cationic polymer and DNA. Therefore, the threshold N/P ratios of 4.38:1 for PDMAEMA + DNA and 2.6:1 for PEI + DNA (Supplementary Figure S3) were chosen for the DNase I degradation experiments. For DNase I protection assay, DNase I (0.1 mg/mL) was added to the same amount of DNA (1 μg) combined with PDMAEMA or PEI at the threshold N/P ratio. The naked DNA was degraded by DNase I (Lane 4, Figure 2B) as the control group and was used to compare to the PDMAEMA + DNA and PEI + DNA groups to test the protection of PDMAEMA and PEI. The DNase I protection assay showed that PDMAEMA and PEI were capable of forming compact complexes with DNA, which could largely protect DNA from nuclease degradation (Lane 6 compared with Lane 5, Lane 8 compared with Lane 7, Figure 2B). A part of the DNA from PEI + DNA was not shifted but retained in the sample well, and the unclear band at the bottom of the lanes was the degraded DNA (Lane 8, Figure 2B). The more amount of degraded DNA means the weaker ability of cationic polymers to protect DNA from degradation. The protective ability of PDMAEMA was even stronger than that of PEI (Lane 6 compared with Lane 8, Figure 2B). Similar results about DNase I protection assay of the peptide dendrimers were shown in the previous study (Luo et al., 2011).

### Transfection in Isolated *Arabidopsis thaliana* Protoplasts

**FIGURE 3:**
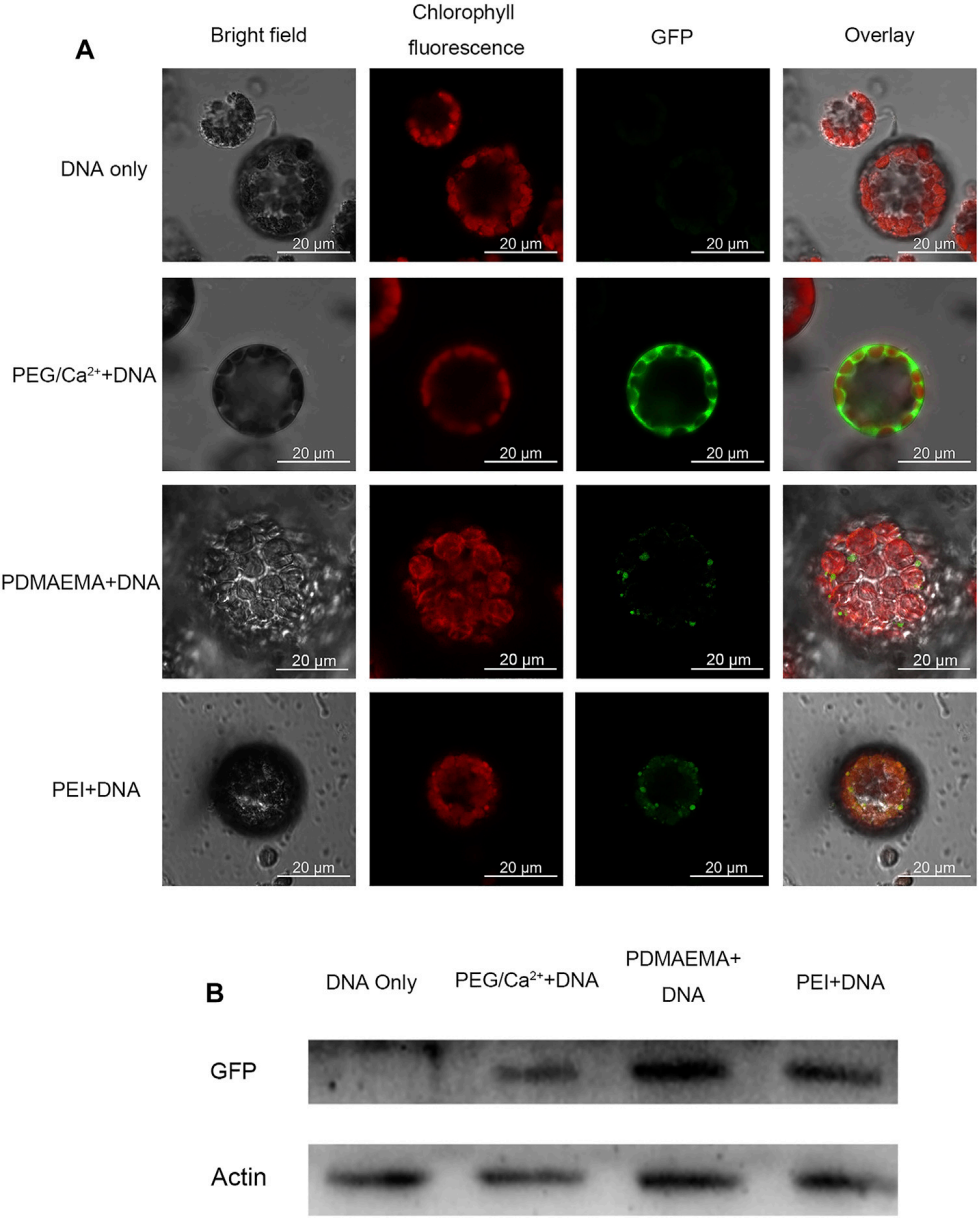
GFP expression imaging in *Arabidopsis thaliana* protoplast. **(A)** Confocal microscope was used to image the *Arabidopsis thaliana* protoplast incubated with DNA only as a negative control, PEG/Ca^2+^ as a positive control, PDMAEMA + DNA (N/P ratio of 15:1), and PEI + DNA (N/P ratio of 15:1). GFP fluorescence images were captured with 488 nm laser excitation and 539 nm emission wavelengths. Chlorophyll autofluorescence images were captured with 633 nm laser excitation and 691 nm emission wavelengths. Scale bar, 20 μm. Protoplasts were incubated with PEG/Ca^2+^ for 15 min and washed with W5 solution to remove PEG. Then, protoplasts were resuspended with W5 solution in a 1.5-mL tube for another 12 h in the dark. The protoplasts were incubated with PDMAEMA + DNA or PEI + DNA directly for 12 h. **(B)** Western blot of the GFP expression level of *Arabidopsis thaliana* protoplasts incubated with DNA only, DNA-PEG/Ca^2+^, PDMAEMA + DNA, and PEI + DNA.

Generally, the cell wall of plants consists mostly of fibrous structures such as cellulose, pectin, and lignin, which may play a role of barrier against the entry of exogenous substances. Protoplasts are plant cells without cell walls. Therefore, to avoid the cell wall as a barrier, the protoplast isolated from *Arabidopsis thaliana* leaves was employed to perform the transfection experiment. Plasmid DNA (pBI221 vector contains a GFP gene) was used as a report gene in the transfection experiment. The total charges of PDMAEMA + DNA and PEI + DNA complexes maintained a positive value (Supplementary Figure S4). Hence, it facilitated the interaction with negatively charged cell membranes and the internalization of complexes into plant protoplasts (Lakshmanan et al., 2013). To evaluate the transfection of PDMAEMA and PEI, DNA complexes of PDMAEMA or PEI were incubated with protoplasts isolated from *Arabidopsis thaliana* leaves as an expression host. Transient GFP expression was detected in protoplasts when protoplasts were incubated with PDMAEMA + DNA or PEI + DNA 24 h later, while there was no GFP fluorescence observed in the protoplasts treated with DNA only (Figure 3A, Supplementary Figure S4).

Polyethylene glycol 4,000 (PEG 4000) is a polymer fusogenic agent, which is commonly used for plant protoplast transfection. It can facilitate the entry and expression of DNA by changing membrane permeability with the existence of Ca^2+^ (Yoo et al., 2007) and was used as a positive control in this experiment. Western blot was also performed to verify the GFP expression in the protein level in the protoplasts incubated with PDMAEMA + DNA, PEI + DNA, and PEG/Ca^2+^+DNA (Figure 3B). The GFP fluorescence of control PEG/Ca^2+^+DNA was obviously stronger than that of PDMAEMA + DNA and PEI + DNA, but the Western blot band of the control was weaker than that of PDMAEMA + DNA and PEI + DNA. The transfection way of PEG/Ca^2+^ is different from that of PDMAEMA + DNA and PEI + DNA. Therefore, the incubation time between protoplast and PDMAEMA + DNA or PEI + DNA was 12 h while that of PEG/Ca^2+^ was 15 min. The more fluorescence but less protein level for PEG/Ca^2+^+DNA treatment may be caused by the protoplast buffer containing PEG 4000, which was not exactly removed in the blocked sample running in one way. Also, we used all the protoplast solution to run the Western blot instead of the supernatant. The results demonstrated that the PDMAEMA + DNA and PEI + DNA can serve as delivery systems and cause DNA expression in *Arabidopsis thaliana* protoplasts.

### Transfection in *Arabidopsis thaliana* Leaves

*Arabidopsis thaliana* leaves were used as the experiment material to further study the expression of plasmid DNA delivered by these two polymers. After preparation of PDMAEMA + DNA and PEI + DNA at the N/P ratio of 15, they were infiltrated on the abaxial surface of the three-week-old *Arabidopsis thaliana* leaves lamina using a needleless syringe. DNA only (in 10 mM MgCl_2_/MES) was used as a control. GFP fluorescence was observed by confocal microscopy in the stoma cells of leaves, 24 h after infiltration with these two complexes. There was no GFP fluorescence detected in the leaves infiltrated with DNA only (Supplementary Figure S5). These results showed that PDMAEMA + DNA and PEI + DNA traverse the plant cell wall and the plasma membrane to enter the plant cell in some way and have an expression in the stoma cells of leaves within 24 h. But, this transfection effect is not so significant compared to that of protoplasts. One possible reason was that infiltration time is not sufficient for a preferable expression. Therefore, a time-course transformation experiment was performed in a subsequent study.

### Transfection in *Nicotiana benthamiana* Leaves

**FIGURE 4:**
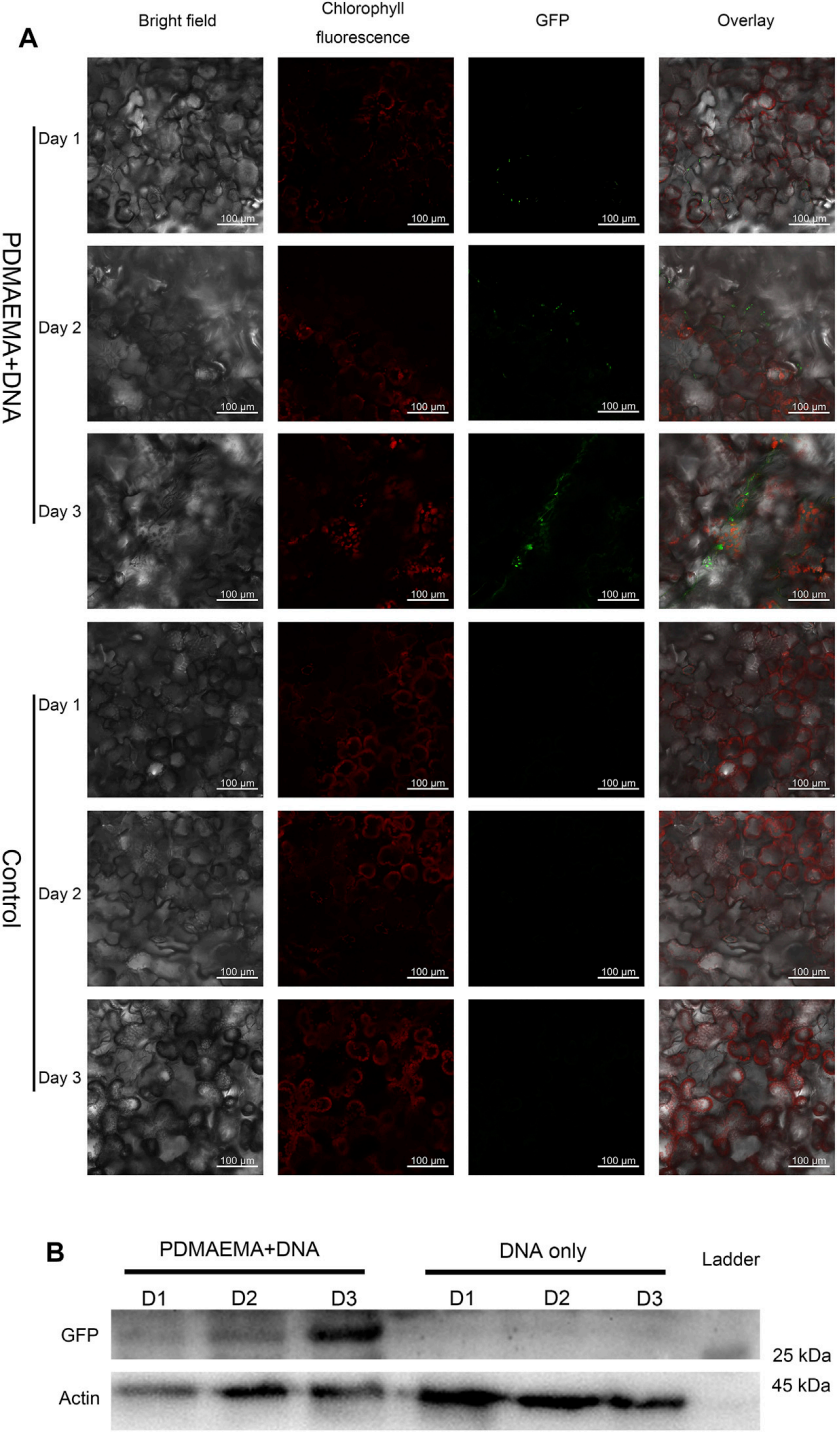
GFP expression in *Nicotiana benthamiana* leaves. **(A)** *Nicotiana benthamiana* leaves infiltrated with DNA only (in 10 mM MgCl_2_/MES as a control) and PDMAEMA + DNA (N/P ratio of 15:1) are imaged using a confocal microscope to detect GFP expression in the leaf lamina in 1 day, 2 days, and 3 days. Experiments were performed with intact leaves from healthy plants. Scale bar, 100 μm. **(B)** Western blot of GFP expression of *Nicotiana benthamiana* leaves infiltrated with DNA only (in 10 mM MgCl_2_/MES as control) and PDMAEMA + DNA in 1 day, 2 days, and 3 days.

Additionally, gene expression experiments were also performed in *Nicotiana benthamiana* leaves to demonstrate the applicability of cationic polymers in different plant species. *Nicotiana benthamiana* leaves of three weeks old were infiltrated with DNA only (in 10 mM MgCl_2_/MES with pH 5.7 as control) and PDMAEMA + DNA on three successive days. On the fourth day, one part of leaves of different treatment was collected to perform confocal microscope imaging (GFP expression for 1 day, 2 days, and 3 days was observed at the same time), while the other part of the leaves was collected to perform Western blot to detect GFP expression in the protein level. The cells around the infiltrated area were transfected with PDMAEMA + DNA (Figure 4A) or PEI + DNA (Supplementary Figure S6A), and GFP fluorescence was observed in both the leaves treated with PDMAEMA and PEI compared to the autofluorescence of chloroplasts (red in Figure 4A) as control. Specifically, the DNA complexes of PDMAEMA showed a GFP expression on first day, and the transfection effect was gradually obvious up to the third day (Figure 4A). The time-course study showed that the PDMAEMA-based gene delivery system was effective to perform transfection in a short time, and the PEI-based gene delivery system had a similar phenomenon (Supplementary Figure S6A). For control experiments, transfection into *Nicotiana benthamiana* leaves was performed using DNA in 10 mM MgCl_2_/MES, and there was no GFP fluorescence detected in the control group (Figure 4A). Western blot was also performed to confirm the GFP expression in the case of time-course treatment (Figure 4B, also see Supplementary Figure S6B), and it was basically consistent with confocal image results.

Based on the transfection results related to *Nicotiana benthamiana* leaves, the transfection behavior with PDMAEMA was achieved in both *Arabidopsis thaliana* and *Nicotiana benthamiana* leaves. The internalization mechanism of complexes is via endocytosis pathways in animal cells (Midoux et al., 2008). But, the specific internalization mechanism of exogenous complexes remains unknown in plant cells (Xia et al., 2021). The endocytotic pathway in plant cells is different from that in the animal; during the process, the pH of the endosomes does not decrease to a dangerous level. Rather, the vacuole of plant cells possesses the lowest pH and is the final destiny for endocytosis (Xia et al., 2020). Plant cell walls consist of some modifications such as pit, perforation plate, spiral thickening, and wart. Also, the diameter of some pits is more than 1 μm, so a minority of complexes may enter the cell via pits of the cell walls (Lakshmanan et al., 2013). Besides, there is ample evidence for stomatal uptake of nanoparticles, for example, hydrophilic chitosan nanocarriers (86.8 nm in size) entered through stomata in the plant leaves (Nadendla et al., 2018). PEI is a cationic polymer with a proton sponge effect. PEI as a carrier is widely used in the delivery of nucleic acids in animal cells (Godbey et al., 2001), and PDMAEMA may have a similar delivery function.

**FIGURE 5:**
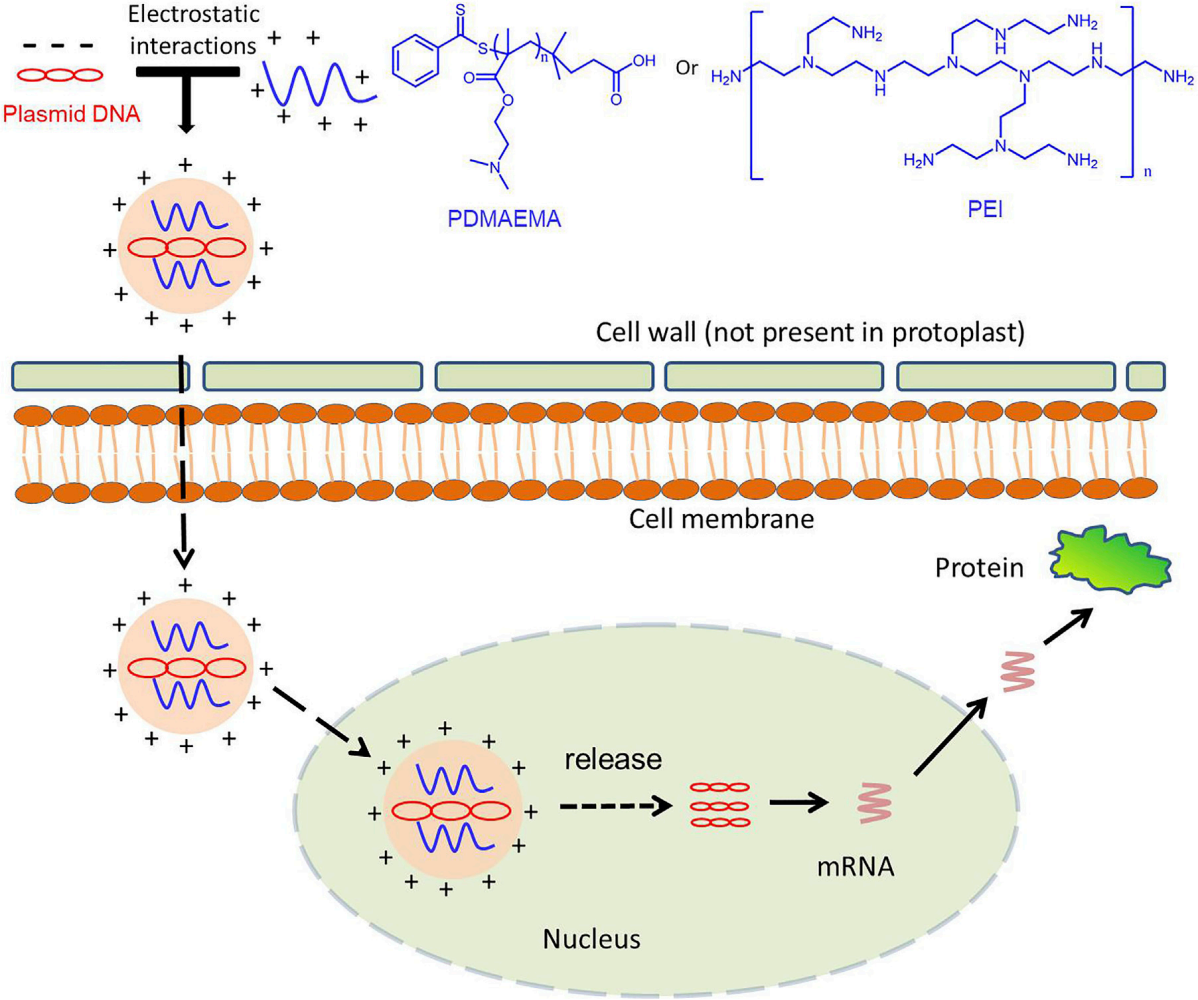
Schematic illustration for the polymer-based gene delivery system in the plant cell.

**SCHEME 1:**
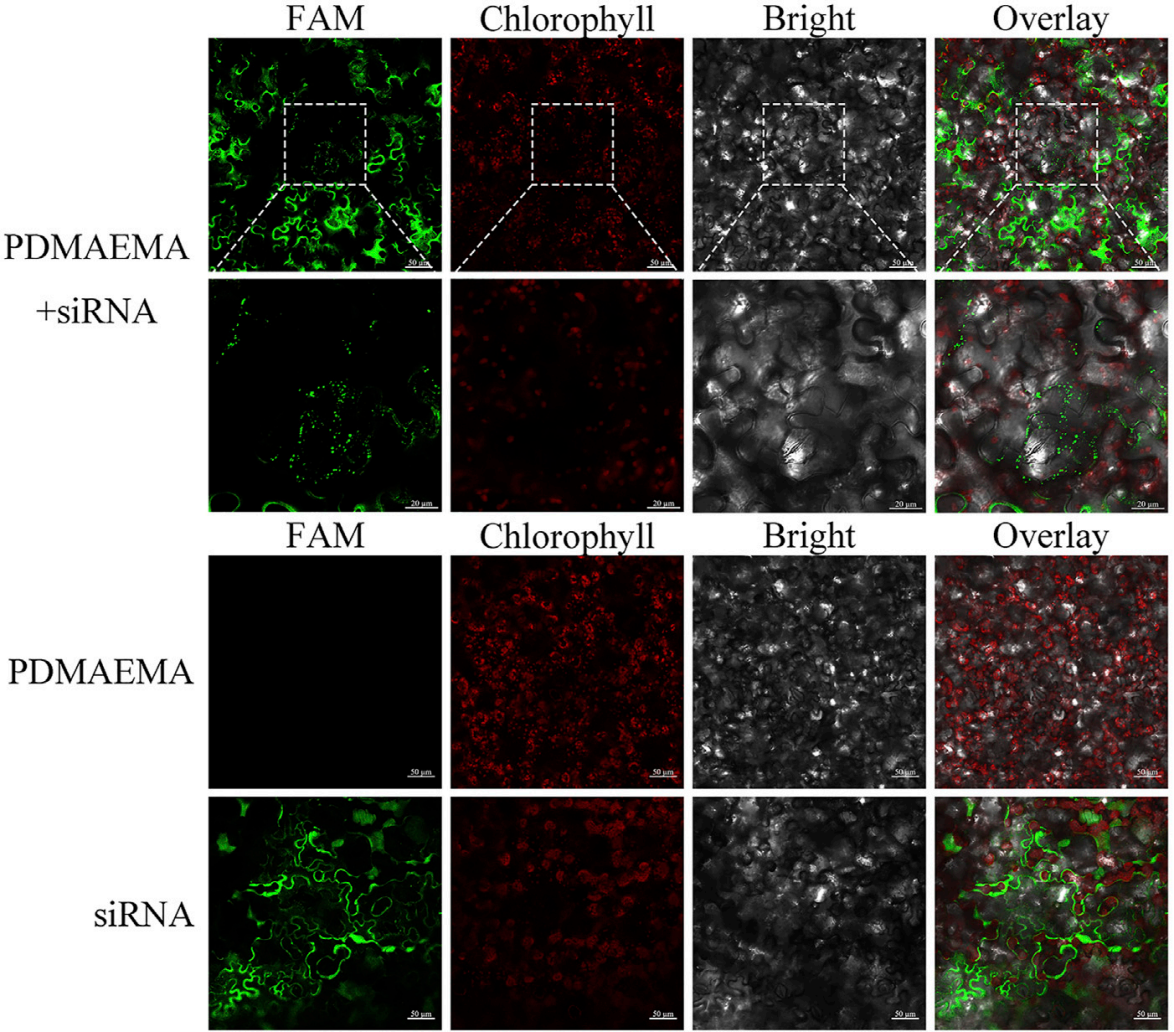
PDMAEMA + FAM-siRNA_*NPR1*_-infiltrating *Nicotiana benthamiana* leaves. FAM-siRNA_*NPR1*_ (20 μM) was mixed with PDMAEMA (1 mg/mL) at the fixed N/P ratio of 15 and incubated at room temperature for 30 min. The formed complexes were diluted 10 times in 10 mM MgCl_2_/MES (pH 5.7) before infiltration, taking only infiltrating PDMAEMA or FAM-siRNA_NPR1_ as a control.

To verify the delivery function of PDMAEMA, we used the complex of FAM-siRNA_*NPR1*_ and PDMAEMA to infiltrate *Nicotiana benthamiana* leaves and observed its localization by confocal microscopy. Because PDMAEMA binds stably to FAM-siRNA_*NPR1*_, the fluorescence of FAM represents the entry of PDMAEMA into cells. Point-like distribution and clustering were observed, but only infiltrating FAM-siRNA_*NPR1*_ did not have this phenomenon; these results illustrated that PDMAEMA was able to deliver nucleic acid into plant cells (Figure 5). According to the article (Midoux et al., 2008) and our results, we proposed a scheme: plasmid DNA interacts with cationic polymers by electrostatic interaction forming positively charged complexes. Then, the positively charged complexes make it easy to traverse the plant cell wall and interact with the membrane to internalize into the plant cell. After entering the plant cell, PDMAEMA and PEI can also protect DNA from degradation. Most of the complexes release plasmid DNA through the proton sponge effect, and it is possible that some DNA enters the nucleus and participates in the transcription and translation. A small part of the complexes enters the nucleus and releases plasmid DNA complexes with an unknown mechanism, participating in the transcription process and translation (Scheme 1). The specific mechanism deserves further study.

### Toxicity Tests in Plant Leaves

To evaluate biocompatibility of these two polymers, we performed toxicity assays in *Nicotiana benthamiana* leaves. We use the expression of *respiratory burst oxidase homolog B* (*Nbrboh B*), a known gene indicating stress conditions in *Nicotiana benthamiana*, to quantify the toxicity of the polymers as they are directly proportional (Demirer et al., 2019). We used qPCR analysis to determine the toxicity of these two polymers at different time points of the treatment.

**FIGURE 6:**
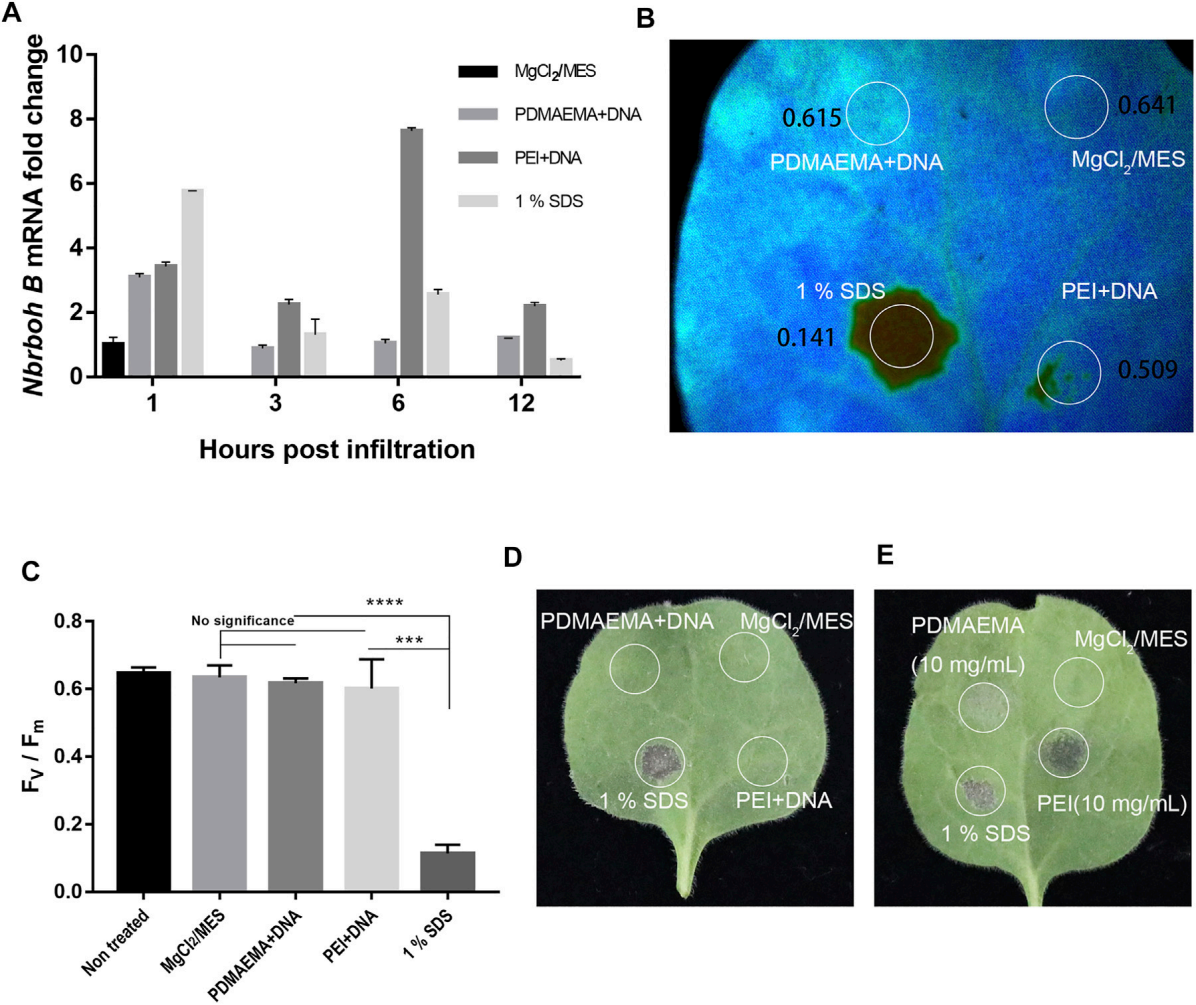
Toxicity assay. **(A)** qPCR analysis of *Nbrboh B* in *Nicotiana benthamiana* leaves with different infiltrations (MgCl_2_/MES (10 mM) as control, PDMAEMA + DNA (N/P ratio of 15:1), PEI + DNA (N/P ratio of 15:1), and 1% SDS at a time course. **(B)** Photosystem II Fv/Fm of different locations treated with PDMAEMA + DNA (N/P ratio of 15:1), PEI + DNA (N/P ratio of 15:1), 1% SDS, and MgCl_2_/MES (10 mM) as control on the same *Nicotiana benthamiana* leaf. **(C)** Statistical data of the (B) Fv/Fm ratio represent the variable/maximum fluorescence measurement of the photosystem II quantum efficiency. **(D)** Phenotype of the single *Nicotiana benthamiana* leaf infiltrated with MgCl_2_/MES (10 mM), PDMAEMA + DNA (N/P ratio of 15:1), PEI + DNA (N/P ratio of 15:1), and 1% SDS on different positions. **(E)** Phenotype of a single *Nicotiana benthamiana* leaf infiltrated with MgCl_2_/MES (10 mM), 1% SDS, PDMAEMA (10 mg/mL), and PEI (10 mg/mL) on different positions.

As shown in Figure 6A, at 1 h, samples treated with PDMAEMA + DNA and PEI + DNA upregulated *Nbrboh B* related to the sample treated with 10 mM MgCl_2_/MES (as a negative control). However, they did not upregulate *Nbrboh B* too much compared to the sample treated with 1% SDS (as a positive control). These results illustrated that 1% SDS caused the most toxicity; at 3 h, 1% SDS, PDMAEMA + DNA, and PEI + DNA caused the upregulation of *Nbrboh B* to decrease, and the expression of PEI + DNA treatment was the most and showed that the toxicity of PEI may be the largest; *Nbrboh B* was at the highest level at 6 h and 12 h with PEI + DNA treatment, while PDMAEMA + DNA treatment with *Nbrboh B* was a continuous downward process; hence, the toxicity of PDMAEMA might be lower than that of PEI. It was in accordance with the previous study that PDMAEMA had less toxicity than PEI does (Verbaan et al., 2003). There are reports that *RbohB* is a fast responsive gene that can be upregulated within minutes; therefore, the long sampling time points are not quite reasonable for the purpose of the experiment (Xia et al., 2020), so the high expression level for PEI at 6 h could result from a second wounding wave for stressed plants, and 12 h is too long to sample for *RbohB* genes.

Additionally, photosystem II measurements were also performed to determine the toxicity in *Nicotiana benthamiana* leaves (Figure 6B). The results showed that PDMAEMA + DNA-infiltrated positions (with an Fv/Fm ratio of 0.615) and PEI + DNA-infiltrated positions (with an Fv/Fm ratio of 0.641) in *Nicotiana benthamiana* leaves had similar photosynthesis quantum yields. But, position treated with 1% SDS as a positive control showed a significant decrease in the photosystem II quantum yield with an Fv/Fm ratio of 0.141 in *Nicotiana benthamiana* leaves, indicating a strong stress or tissue damage. Also, there was no significant difference between the position treated with 1% SDS and the negative control (Figure 6C). The phenotype of positions with different infiltration of leaves showed that PDMAEMA + DNA and PEI + DNA had no obvious toxicity compared with 10 mM MgCl_2_/MES treatment as a negative control and 1% SDS treatment as a positive control (Figure 6D).

Furthermore, in order to compare the toxicity between PDMAEMA and PEI, we used the diluted PDMAEMA (10 mg/mL) and PEI (10 mg/mL) in 10 mM MgCl_2_/MES and infiltrated the *Nicotiana benthamiana* leaves. PEI showed a higher toxicity than PDMAEMA according to the phenotype of *Nicotiana benthamiana* leaves (Figure 6E). The result was consistent with the qPCR analysis (Figure 6A) and previous examinations toward animal cells (Verbaan et al., 2003; Xu et al., 2009; Agarwal et al., 2012).

## Conclusion

The gene delivery system on the basis of cationic polymer PDMAEMA is an original method for transient transformation in plant cells. In this proof-of-concept study, complexes of DNA and cationic polymers (PDMAEMA and PEI) with a small hydrodynamic diameter were formed at the specific N/P ratio. Meanwhile, we confirmed that PDMAEMA and PEI protect DNA from degradation after the formation of complexes (PDMAEMA + DNA and PEI + DNA). The expression of pGFP was detected by confocal microscopy imaging and confirmed by Western blotting analysis. Polymer-mediated delivery is appropriate to transient transfection because of its operability efficiency and low toxicity. In conclusion, PDMAEMA-mediated transient transformation is achieved in plant cells without obvious toxicity or tissue damage. PDMAEMA and PDMAEMA-based gene delivery materials are more promising for gene delivery to plants due to their relatively low cytotoxicity and facile fabrication compared to PEI. Polymer-based plant transfection platforms probably provide promising extra opportunities in plant genetic engineering.

## Data Availability

The datasets presented in this study can be found in online repositories. The names of the repository/repositories and accession number(s) can be found in the article/Supplementary Material.
