# Supplementary material for: Efficient Transient Expression of Plasmid DNA Using Poly (2-(N,N-Dimethylamino) Ethyl Methacrylate) in Plant Cells
Source: Front Bioeng Biotechnol. 2022 Feb 22;10:805996. doi: 10.3389/fbioe.2022.805996 (PMC8902165; doi:10.3389/fbioe.2022.805996)
Supplement: Supplementary file 1 [file Presentation1.pdf]

Supplementary Figures

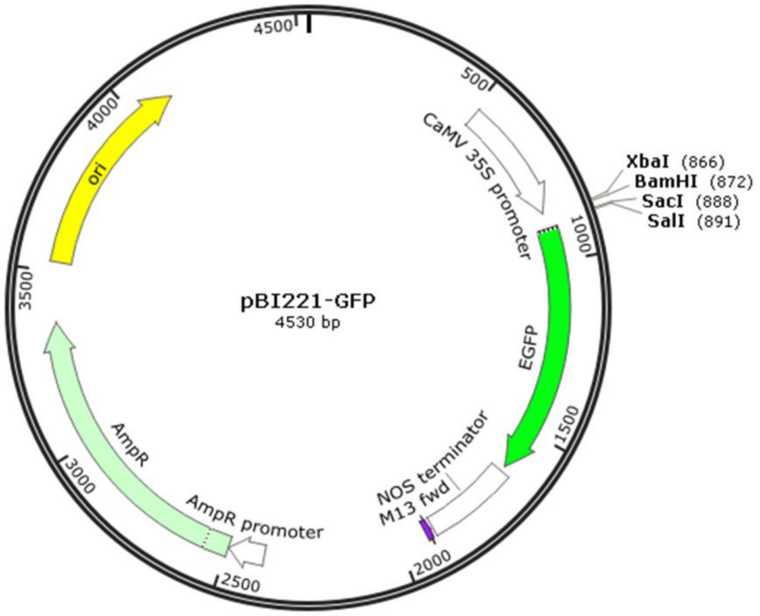

Supplementary Figure 1. Map of plasmid DNA pBI221-GFP.

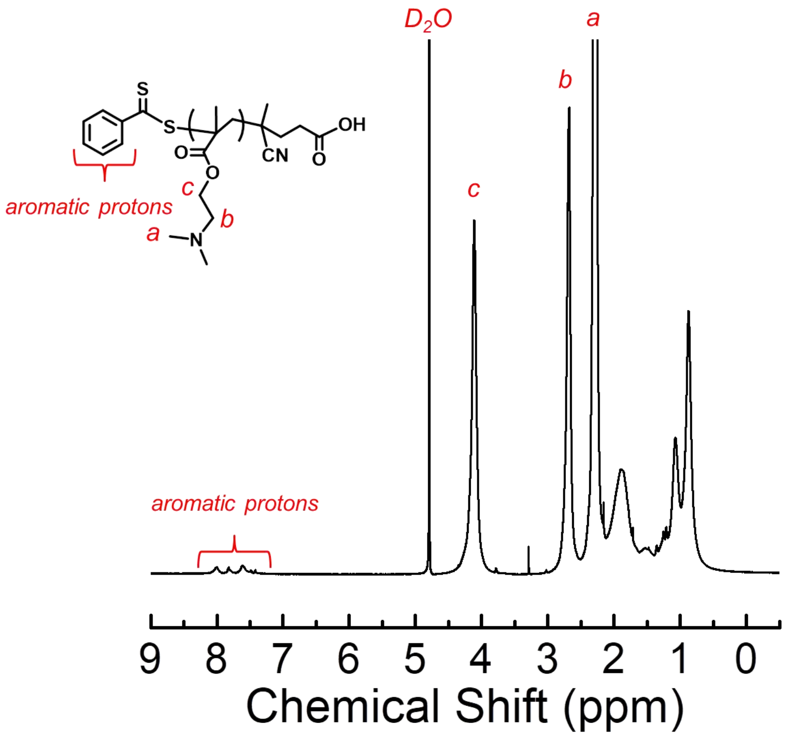

Supplementary Figure 2. [1]H Nuclear Magnetic Resonance spectrum of $PDMAEMA_{37}$ in *D_2O*.

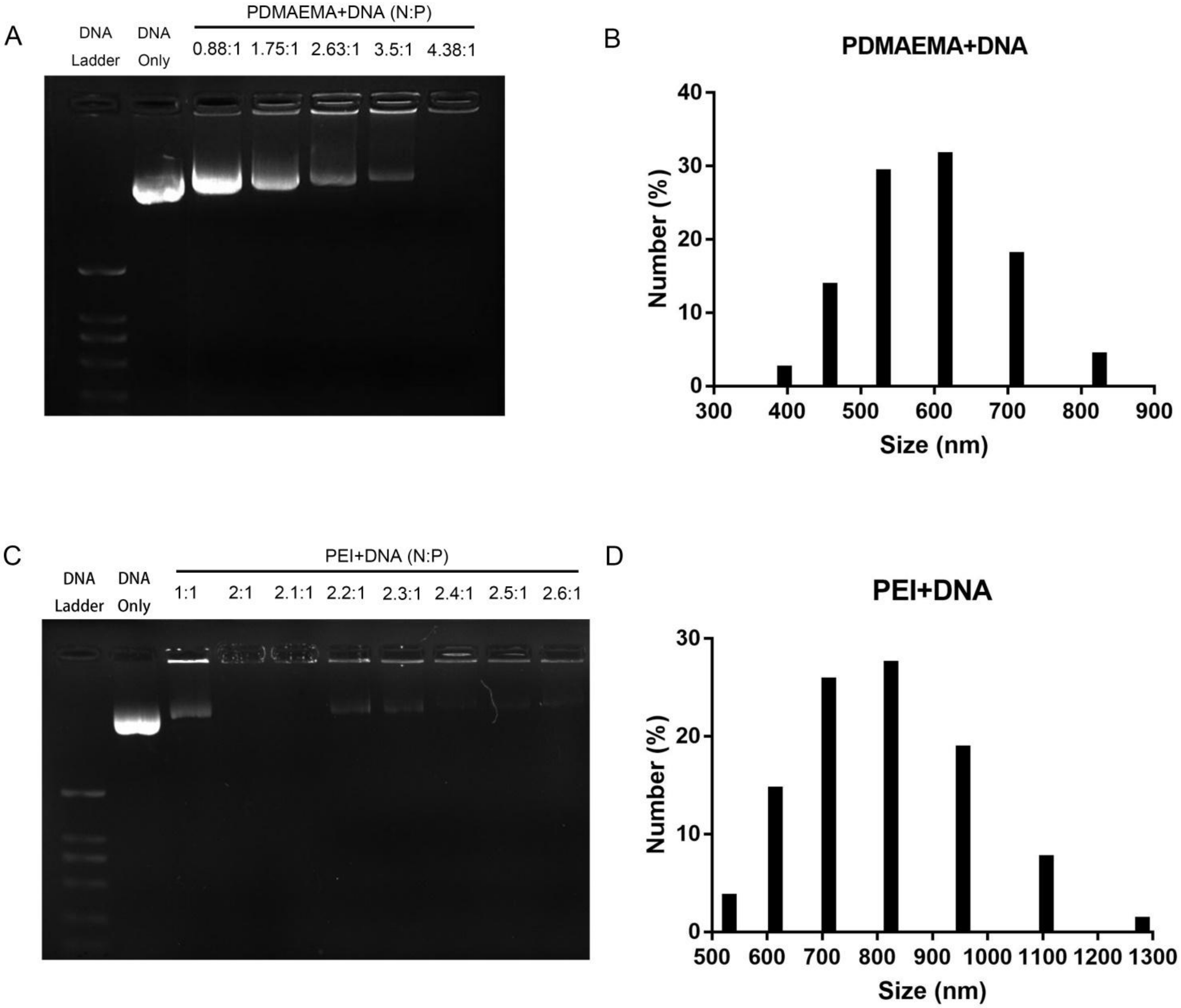

Supplementary Figure 3. Gel retardation assay. (A) Gel retardation assay of PDMAEMA+DNA (1 μg) at relatively low N/P ratios of 0.88:1, 1.75:1, 2.63:1, 3.5:1 and 4.38:1. (B) Hydrodynamic diameter distributions of PDMAEMA+DNA (5 μg) at N/P ratio of 4.38:1. (C) Gel retardation assay of PEI+DNA (1 μg) at relatively low N/P ratios of 1:1, 2:1, 2.1:1, 2.2:1, 2.3:1, 2.4:1, 2.5:1 and 2.6:1. (D) Hydrodynamic diameter distributions of PEI+DNA (5 μg) at N/P ratio of 2.6:1.

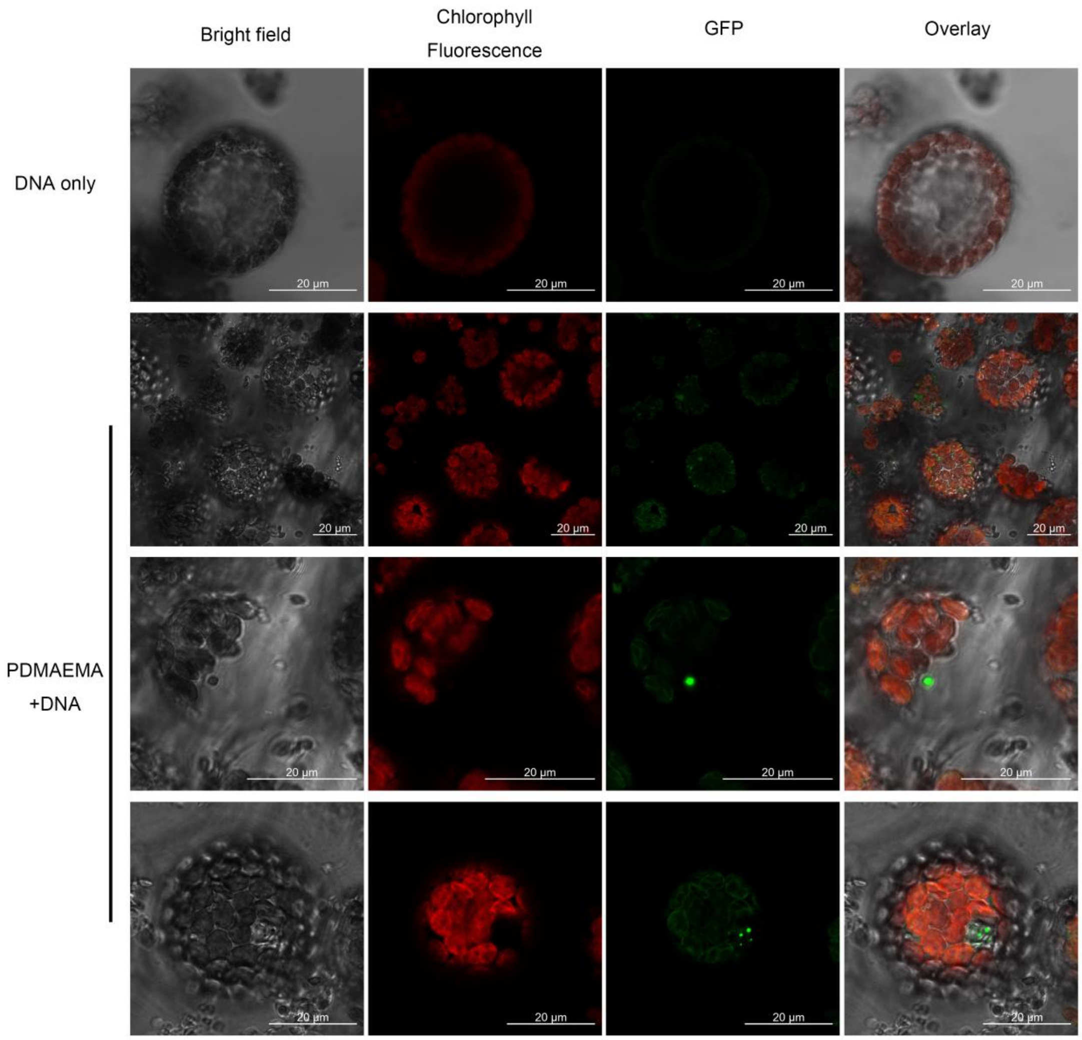

Supplementary Figure 4. CLSM images of *Arabidopsis thaliana* protoplasts after incubation with PDMAEMA+DNA (N/P ratio of 15) for 24 h. Scale bar, 20 µm.

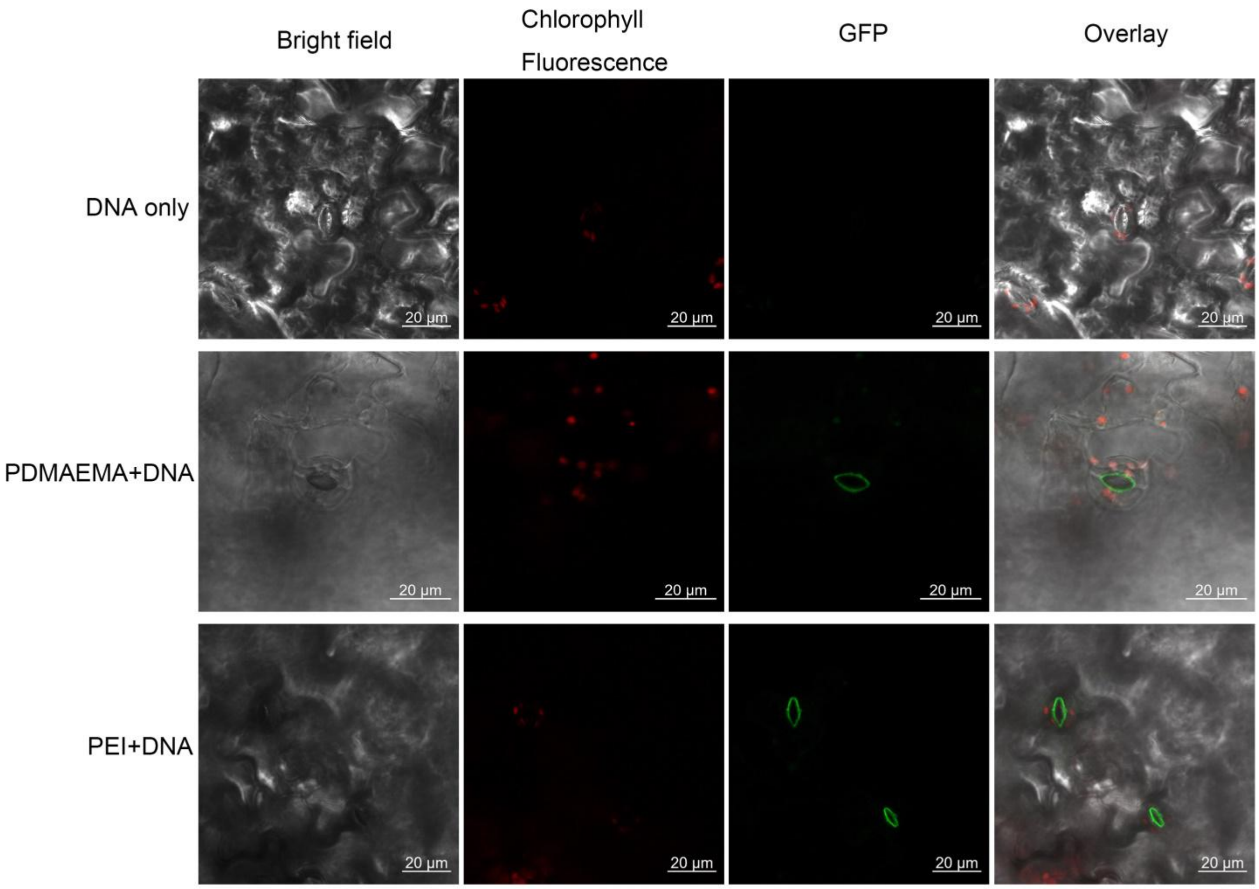

Supplementary Figure 5. GFP expression imaging in *Arabidopsis thaliana* leaves. Wild-type *Arabidopsis thaliana* leaves infiltrated with DNA only (in 10 mM $MgCl_2$/MES as control), PDMAEMA+DNA (N/P ratio of 15) and PEI+DNA (N/P ratio of 15) are imaged with confocal microscope to detect GFP expression in the leaf lamina in 24 h. Experiments were performed with intact leaves from healthy plants. Scale bar, 20 µm.

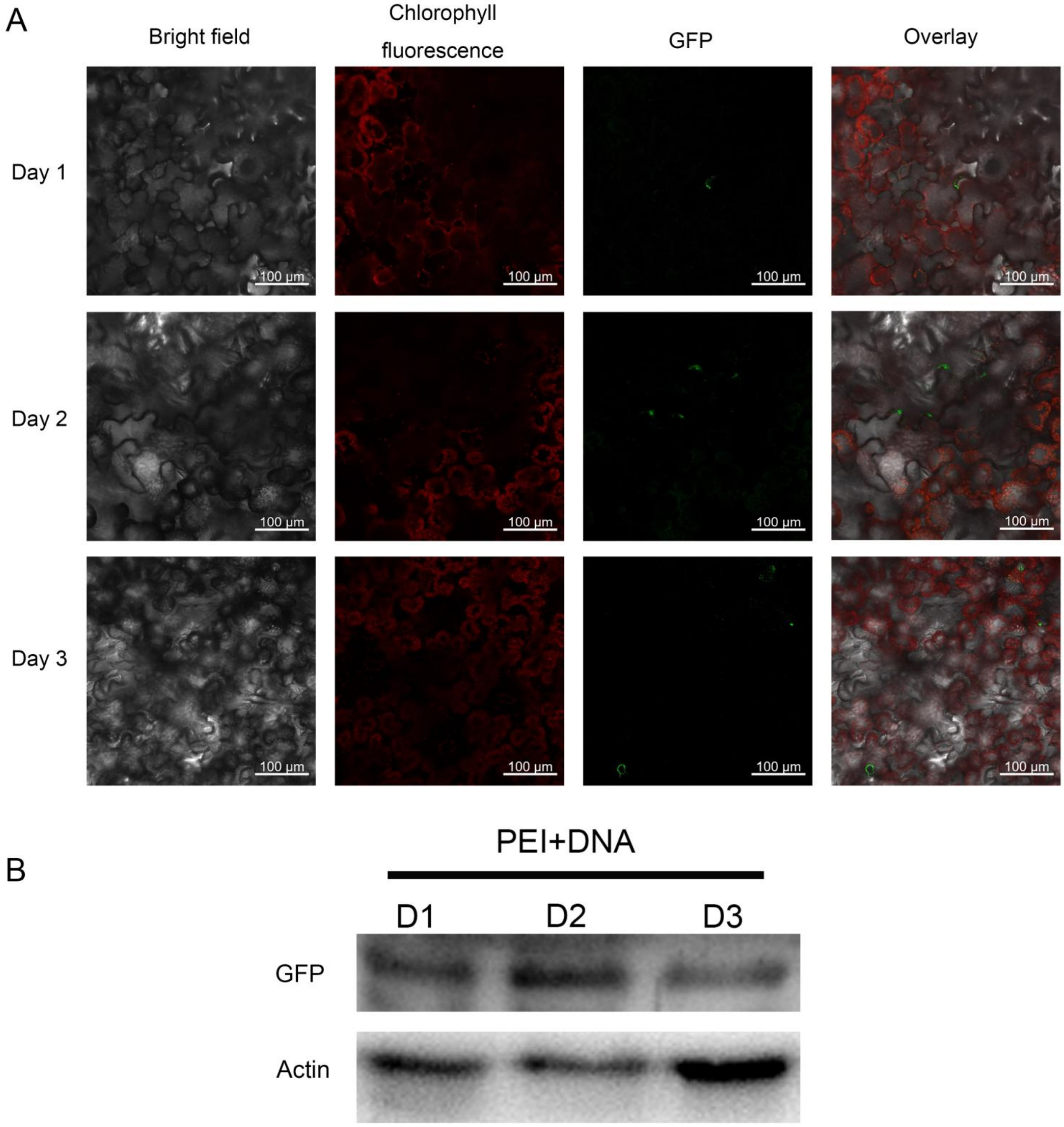

Supplementary Figure 6. GFP expression in *Nicotiana benthamiana* leaves infiltrated with PEI+DNA. (A) GFP images of *Nicotiana benthamiana* leaves infiltrated with PEI+DNA in day 1, day 2 and day 3. Images of DNA only (in 10 mM $MgCl_2$/MES as control) as control treatment are shown in Figure 4A. Scale bar, 100 µm. (B) Western blot of GFP expression of *Nicotiana benthamiana* leaves infiltrated with PEI+DNA in 1day, 2 days and 3 days. Western blot of DNA only (in 10 mM $MgCl_2$/MES as control) as control treatment is shown in Figure 4B.
